# Supplementary material for: Are carbon emissions trading and green financial instruments synergistic? -Comprehensive quantitative research based on content analysis
Source: PLoS One. 2024 Mar 7;19(3):e0298601. doi: 10.1371/journal.pone.0298601 (PMC10919601; doi:10.1371/journal.pone.0298601)
Supplement: S1 Data — (ZIP) [file pone.0298601.s001.zip › Data/地级市Z_F工作报告.zip!/╣π╬≈/╖└│╟╕█╩╨/└┤╫╘╣1⁄2╓┌║┼í╛┬φ┐╦╩2╛▌═°í┐_2016.docx]

各位代表：

　　现在，我代表市人民政府，向大会报告市五届人民政府过去五年工作，对今后五年工作提出建议，请予审议，并请政协委员和其他列席人员提出意见。

　　过去五年工作回顾

　　本届政府履职五年来，在自治区党委、政府和市委的正确领导下，在市人大、市政协的监督和支持下，团结带领全市各族人民，始终把发展作为第一要务，主动适应经济发展新常态，不断完善发展思路，抢抓机遇，敢做善成，开创了改革开放发展新局面，较好地完成了本届政府各项目标任务。

　　这五年，我们坚持开拓创新，砥砺前行，综合实力实现赶超跨越。面对国内外复杂形势，不断解放思想，求真务实，克难攻坚，经济保持平稳较快发展态势。全市生产总值先后跨越400、500、600亿元三大台阶，达620.7亿元，年均增长12.1%，总量由全区第13位跃升至第11位，人均GDP突破一万美元，稳居全区首位；财政收入70.6亿元，年均增长15%，人均公共预算收入全区第一；农民人均纯收入突破万元大关；全市规上工业总产值迈上千亿元台阶，达1323亿元，年均增长23.3%，总量跃升全区第八；外贸进出口总额突破80亿美元，年均增长19%；主要经济指标增幅或总量居广西前列，13项指标基本实现翻番。

　　这五年，我们坚持聚焦重点，狠抓项目，发展基础不断夯实。先后组织实施“双百工程”“双二十大事”“十百千”“边海经济带工程包”项目建设计划，累计开工项目1000多项，竣工600多项，完成固定资产投资2440亿元，年均增长13.7%；加强项目策划谋划，储备重大项目400多项，形成开工一批、竣工一批、储备一批的良性循环；推行连片征地拆迁，累计征地8万多亩，落实建设用地指标8.3万亩，有力保障了项目用地需求。引进亿元以上项目169个，重大项目建设不断取得新突破，大发展格局全面拉开。

　　这五年，我们坚持工业强市，转型升级，临港重大产业加快集聚。强化龙头企业带动，培育千百亿元产业，打造千亿元产业园区，推动现代临港产业在做大做强中转型升级。钢铁、有色金属、能源、食品、化工、装备制造等六大支柱产业加速发展，钢铁产业产值突破200亿元，有色金属产业产值突破200亿元，食品产业产值超450亿元。全市园区面积由217平方公里扩展到462平方公里，企沙工业区、大西南临港工业园、高新技术开发区、东湾物流园、跨境经济合作区、冲仑物流园、上思工业集中区等7大园区集聚能力持续增强。中国西部首个核电站红沙核电并网发电，“华龙一号”三代核电技术示范机组开工建设。金川铜冶炼、钢铁冷轧建成投产。全市规上工业企业162家，其中产值超亿元企业126家，百亿元级企业4家。盛隆公司跻身中国五百强企业，成为广西民营企业“一哥”，大海粮油、惠禹粮油成为广西百亿元强优工业企业。我市成为全国最大的植物油籽、磷酸加工出口基地和西部重要的新能源基地。

　　这五年，我们坚持开放带动，先行先试，边海开放新格局初步确立。主动对接国家“一带一路”和自治区“双核驱动”战略，面向东盟的对外开放格局加快形成。与全球190多个国家和地区保持贸易往来，累计完成外贸进出口额274亿美元，边贸年成交额迈上300亿元台阶，边贸企业达600多家，口岸出入境人数累计超过2000万人次。东兴试验区、跨境经济合作区、沿边金融综合改革试验区、构建开放型经济新体制综合试点城市、边境经济合作区、跨境旅游合作区等开放平台加快建设。敢于先行先试，创造了5个“全国第一”、15个“广西第一”。加快口岸“三个一”及通关一体化改革，国际贸易“单一窗口”率先在全区上线运行。成功承办第七届中国-东盟社会发展与减贫论坛、“魅力东盟·走入中国”文化之旅、中越边民大联欢、中越跨境经济合作论坛等十多次重大国际活动。

　　这五年，我们坚持彰显特色，完善功能，生态海湾城市初显魅力。拉开城市大框架，完善城市配套，城市面貌日新月异。城市建成区扩大到55.2平方公里，人口城镇化率达55.1%，居广西前列。大手笔推进海湾新区建设，东湾大道、西湾大道、文昌大道、针鱼岭大桥建成通车，加快建设防城至江山一级路、江山半岛环岛东路，完善中心区、桃花湾片区、城南新区等片区路网，“五纵五横”城市路网基本形成。海洋文化公园等4大公园和科技馆等7大场馆全面开放。新建供水管网153公里，完成城区双回路供水管网改造。中缅天然气管道防城港段建成，城区燃气普及率85%。累计销售商品房870万平方米，增长2.7倍。成立城市管理综合执法局，城市精细化管理水平显著提高。完成北部湾大道等主干道景观亮化工程，西湾绿道投入使用。全市森林面积35.8万公顷，森林覆盖率63%，城镇污水集中处理率85%以上。空气质量优良率94.2%，主要水源水质优良率100%，空气和水源质量指数广西第一。“美丽乡村”建设成效显著，农村人居环境明显改善。先后获得“中国氧都”“中国长寿之乡”“中国白鹭之乡”“中国最美休闲乡村”“中国美丽田园”“广西园林城市”“自治区卫生城市”等荣誉称号。荣居中国最适宜养老城市19位，中国宜居城市竞争力36位。

　　这五年，我们坚持以港立市，交通先行，区域性国际枢纽加快形成。制定5亿吨、500万标箱大港建设计划，推动综合交通基础设施建设，建成西部第一个亿吨大港。提升渔万港区吞吐能力，启动企沙港区建设，累计完成港口投资124.2亿元，开工建设深水泊位18个，建成9个，万吨级泊位达36个。20万吨级进港航道、粮食等一批专用泊位投入使用，新增集装箱通过能力135万标箱，港口吞吐能力达1.5亿吨。完成沿海铁路防城港段扩能改造，开通南防高铁，我市迈入高铁时代。建成防东高速、钦崇高速上思段，在全区率先县县通高速，1小时交通圈基本形成。

　　这五年，我们坚持创新驱动，激发活力，发展新优势日益彰显。强化科技创新，建成省部级重点实验室1家、工程技术研究中心4家，新增17家广西技术创新示范企业、23个广西名牌、16件广西著名商标。新建科技企业孵化器6个，入孵企业超百家。高新技术企业达12家，产值突破200亿元，年均增长40.7%。每万人发明专利拥有量从0.08件提升至1.39件。推动“互联网+双创”，建成“IT小镇”门户，启动云朗科技园建设。防城港·阿里巴巴进口货源平台成功上线。全市物流企业达400多家，东湾物流园区成为广西首家国家级示范物流园区。进境水果快速航线实现常态化运行，开通防城港至北京等地的冷链专列和海铁联运五定班列，海陆联动的冷链产业加快形成。新增金融机构44家，设立广西首支地级市产业基金，27家企业进入自治区重点培育上市企业库，农村互联网金融蓬勃兴起。全市景区景点达60多个，A级景区达12家，防城港至越南下龙湾海上国际航线复航，中断八年的出入境异地办证业务恢复，东兴口岸启动外国人口岸签证业务，全市累计接待游客人数突破5000万人次，“上山下海又出国”的旅游格局基本形成。

　　这五年，我们坚持全域统筹，融合发展，“三农”工作成效显著。注重城乡协调发展，推进“三农”和县域经济工作。克服“威马逊”等特大自然灾害的影响，现代农业稳步发展，全市创建现代农业示范区9个，发展农业龙头企业38家，建成万元片区25.3万亩、万元村屯600个。打造全国海洋牧场示范基地，水产养殖面积达28.7万亩。实施农民增收工程，农民人均纯收入年均增长13.1%。在全区创新开展乡镇“五化十有”规范化建设，完成“六统强基工程”、村级“十六有”阵地建设。新建改建农村公路1000多公里，率先在全区实现村村通水泥路，89%的乡镇通三级以上公路。县域经济加快发展，上思县主动开放融入，加快绿色崛起，被列入国家社会扶贫创新协作帮扶试点县，“一村一警”等经验在全区示范推广。东兴市充分发挥创新开放平台优势，贸易、旅游、加工制造等产业加快转型升级，城市风貌改造取得较好成效，人口城镇化率接近70%，连续三年位列全国电商百佳县前30强，成为全国首批综合行政执法体制改革试点城市。港口区全力服务全市临港大工业，积极发展国际物流，规上工业总产值占全市七成以上，多次获广西招商引资工作先进单位，簕山古渔村被评为全国休闲农业与乡村旅游示范点。防城区积极融入海湾新区建设，城区南延西拓构架基本形成，商贸物流网络加快打造，一批城市商业综合体建成运营，现代特色农业蓬勃发展，建成全国最大金花茶育苗基地。

　　这五年，我们坚持以人为本，共建共享，民生福祉持续增进。切实改善和保障民生，五年来财政安排民生支出327亿元，增长3.2倍。教育卫生脱贫“三大攻坚”扎实推进。建成20所公办乡镇中心幼儿园和8所高标准城区中小学；广西财经学院防城港学院建成招生，全日制高校建设实现零突破；市特殊教育学校、市高级中学二期建成使用，市理工学校、市北部湾高中加快建设。全市新增学位1.7万个。市第五人民医院、港口区人民医院新院建成启用，市第一人民医院儿科楼、全科医生培训中心等一批项目进展顺利。脱贫攻坚成效显著，累计减少6万多贫困人口。文化建设取得新突破，荣获广西首个“中华诗词之市”，成功获批创建全国公共文化服务体系示范区。人口计生工作扎实推进。城乡居民基本养老保险覆盖率94%，基本医疗保险覆盖率96%。五保户、孤儿、残疾人做到应保尽保。五年新增就业岗位12万个，转移农村富余劳动力10万人，城镇登记失业率控制在2%以内，失业率保持全区最低。改造农村危房22077户，建设保障性住房4.5万套，采取公共租赁住房货币补贴等方式，解决近5万户困难居民的住房问题。安全生产执法监管持续强化，应急处置能力不断提升，食品药品安全专项整治成效明显。加大海洋环境和港口粉尘治理力度，近岸海域水质保持优良。信访工作机制不断创新。严厉打击“两抢一盗”等各类违法犯罪活动，加大反走私、反恐、禁毒力度。抓好边海防管控，国防动员和后备力量建设持续加强，连续四次获“全国双拥模范城”称号。

　　这五年，我们坚持依法行政，简政放权，政府效能进一步提升。开展党的群众路线教育实践、“三严三实”活动和“两学一做”学习教育，激发广大干部群众干事创业活力。严格依法办事，自觉接受人大和政协监督，人大代表建议和政协委员提案办结率100%。深化“放管服”改革，全部取消非行政许可审批事项，行政审批更加规范高效，在全区率先全面公布权力清单和责任清单，市本级41%的审批事项在窗口办结，审批事项日结率达70.8%。成立市仲裁委员会，多元化纠纷解决机制逐步完善。在全区率先启动公安大部门制改革。全国商事制度改革试点成效显著，在广西率先实施“多证合一”。

　　其它各项社会事业全面进步，司法、监察、宗教、侨务、人防、消防、保密、审计、统计、税务、质监、供销、通信、海关、检验检疫、海事、气象、地震、水库移民、农机、社会科学、决策咨询、地方志、档案、法制、妇女儿童、老龄、残疾人、红十字、机关事务等工作取得新进步，工会、共青团、妇联等群团组织在经济社会发展中发挥了重要作用。全市边疆稳定，民族团结，社会和谐，人民安居乐业！

　　各位代表！过去的五年，我们经受了前所未有的考验，取得了令人瞩目的成就。这是自治区党委、政府和市委正确领导的结果，是市人大、市政协监督支持的结果，是全市人民团结奋斗的结果。在此，我代表市人民政府，向全市各族人民，向我市驻军、武警官兵和中直、区直单位，向各民主党派、工商联、无党派人士、人民团体，向关心支持防城港发展建设的港澳台同胞、海外侨胞及海内外朋友们，表示崇高的敬意和衷心的感谢！

　　各位代表，过去五年，我们在历届领导班子打下的坚实基础上，继承中创新、发展中突破，取得了来之不易的成绩，积累了十分宝贵的经验：一是必须扭住发展第一要务不放松，创新发展理念，主动适应新常态，以边海经济带为主线，采取强有力的稳增长措施，全力推进重大产业和项目建设，保持我市经济增长位于全区前列；二是必须坚持开放带动不动摇，实施边海联动战略，敢于先行先试，打造系列开放平台，争当全国沿边开发开放排头兵；三是必须把保障和改善民生放在突出位置，大幅增加民生和社会事业投入，努力为民办实事办好事，保持人均居民收入、人均保障支出广西领先，让全市人民享有更多获得感；四是必须牢固树立绿色发展理念，既要金山银山，也要碧海蓝天，做到开发与保护并重，走出一条符合防城港实际的科学发展之路；五是必须发扬真抓实干的精神，工作要实、标准要高、要求要严，敢于担当，一抓到底抓落实，干一件成一件，各项工作经得起实践的检验。

　　在肯定成绩的同时，也要清醒地看到，我市发展仍然面临不少困难和问题：经济总量还不够大；主导产业不够强，现代服务业发展滞后，高新技术产业仍处在成长期；城市功能配套不够完善，教育卫生等短板依旧突出；城乡区域发展不平衡，脱贫攻坚任务依然繁重；庸政懒政怠政现象不同程度存在，政府建设有待进一步加强。对于这些困难问题，我们必须用新理念新方法加以克服解决。

　　未来五年的奋斗目标和主要工作

　　按照自治区党委、政府统一部署，根据市第六次党代会精神，今后五年市政府工作的总体要求是：高举中国特色社会主义伟大旗帜，以邓小平理论、“三个代表”重要思想、科学发展观为指导，深入贯彻习近平总书记系列重要讲话精神，按照中央“五位一体”总体布局和“四个全面”战略布局，围绕中央赋予广西的“三大定位”和自治区“两个建成”“四大战略”“三大攻坚战”部署，坚持发展第一要务，以提高发展质量和效益为中心，以建设边海经济带为主线，着力推进供给侧结构性改革，全力推进东兴试验区、跨境经济合作区、沿边金融综合改革试验区建设，努力构建引领经济发展新常态的开放型经济新体制，奋力实现打造广西“两个建成”先行区的宏伟目标。

　　今后五年经济社会发展的主要目标是：地区生产总值达1200亿元，年均增长10%，人均地区生产总值达11万元。财政收入达100亿元，年均增长7%。固定资产投资五年累计3500亿元，年均增长10%。规上工业总产值达3000亿元，年均增长18%。外贸进出口总额达140亿美元，年均增长10%以上。居民人均可支配收入达3.43万元，年均增长11%。人口城镇化率达65%，城镇登记失业率控制在4%以内，居民消费价格指数控制在3.5%以内。万元GDP能耗和主要污染物排放控制在自治区下达的指标范围内。

　　实现上述目标，要抓好以下十项重点工作：

　　一、加快边海经济带建设，打造升级发展新引擎

　　突出创新驱动，拓展发展空间，优化产业布局，加快打造边海经济带增长极。

　　强化沿海沿边发展优势。以沿海沿边交通干线为轴，以重点园区为主要节点，形成边海联动、产业集聚的经济发展带。发挥港口、东兴龙头和防城、上思内陆腹地作用，实现县（市、区）产业优势互补。拓展规划发展新空间，落实国家和自治区主体功能区规划、县（市）空间规划，优化企沙半岛、渔万半岛、江山半岛和京族三岛产业布局，建成全市行政文化商务中心、临港工业区、生活居住区、文化旅游集聚发展区。

　　增强发展新动力。以六大业态为重点，实施创新驱动，推动六大业态转型升级。优化港口经济，建设西南最大的港口产业集群；提升口岸经济，加快构建开放型经济新体制试点试验城市；培育旅游经济，推进跨境旅游合作区建设；拓展海洋经济，重视新兴海洋产业的发展，建设海洋经济强市；发展互联网经济，加快“IT小镇”和电子商务跨境交易平台建设；培育生态经济，创建生态文明先行示范区。

　　二、加快工业转型升级，打造现代临港工业体系

　　坚持工业强市，抓住国家实施“中国制造2025”机遇，推动产业向中高端迈进，实现产业集群发展。

　　推动重大产业升级发展。依托龙头企业带动，延伸上下游产业链，发展中高端产业，壮大钢铁、有色金属、能源、食品、石化、装备制造和高新技术等支柱产业，形成竞争优势明显的现代临港工业体系。到2020年，钢铁产业实现产值600亿元，铜镍等有色金属产业700亿元，能源产业200亿元，食品产业600亿元，石化产业200亿元，装备制造和高技术产业600亿元。

　　发展壮大产业园区。整合提升企沙工业区和大西南临港工业园，发展钢铁、有色金属、能源、装备制造、化工及配套产业，力争打造全区首个3000亿元产业园区；抓好东湾物流加工园区国家级示范项目建设，做优粮油食品、石油化工、加工制造和大宗商品物流、冷链物流，打造千亿元产业园区；加快高新技术开发区建设，推进云朗科技园基础配套建设，培育新材料、节能环保、新一代信息技术等新兴产业，打造一批超百亿元的产业集群；推动跨境经济合作区建设，加快布局发展加工贸易、跨境电商、跨境金融等产业；做好企沙东岛能源化工基地前期工作，谋划发展煤-电-热-化、仓储及现代物流等产业。防城工业区重点发展贸易加工、物流、建材等产业；上思工业集中区重点发展蔗糖、朗姆酒、木材加工等产业。完善园区管理体制，增强园区创新发展内生动力。

　　发展海洋经济。建设海工装备制造产业园区，发展海洋船舶、海洋工程装备制造业，壮大海洋交通运输业。合理开发海洋资源，培育海洋生物制药产业。完善企沙渔港配套设施，发展远洋捕捞业，推动现代海洋渔业转型升级。抓好珍珠湾海洋牧场建设，建成国家级海洋牧场示范区。建好江平海洋产业园，提升海产品精深加工能力，打造区域性国际水产交易中心和冷链物流中心。

　　三、加快开放发展，打造开放型经济新体制示范城市

　　以构建开放型经济新体制综合试点试验为契机，敢于先行先试，拓展开放领域，开创面向东盟开放合作新格局。

　　构建开放型经济新体制。抓住我市被列为试点试验城市的重大机遇，按照国家确定的任务要求，落实工作责任，确保大部门制和执法体制、行政审批制度、跨境劳务合作、边民互市贸易转型升级等四项核心改革实现突破，形成一批在全国可复制、可推广的经验。探索开放型经济运行管理新模式、园区协同开放新机制、国际投资合作新方式、质量导向型外贸促进新体系、金融服务开放型经济新举措、全方位开放新格局等事项，构建互利共赢的边海开放型经济新体制。

　　加快开放平台建设。推进东兴试验区升级发展，抓好财税金融、跨境贸易、跨境劳务、跨境旅游等领域创新，扩大先行先试成果。推动跨境经济合作区建设，建好东兴口岸二桥综合服务区国门楼、标准厂房、罗浮西路等一批重点项目，加快冲榄工业园、江平工业园等配套园区建设。深入推进沿边金融综合改革试验区建设，拓展人民币与越南盾可兑换业务，探索建立人民币回流机制，布局建设股权产权交易中心、商品交易所，打造中国-东盟跨境金融中心。

　　推动口岸开放升级。扩大防城港口岸、企沙和江山边地贸口岸开放，申报禽畜、肉类、生猪活体进口等指定口岸。推动峒中口岸升格，加快峒中至横模大桥建设，扩大里火互市区、北仑河二桥开放通道。调整扩大东兴边境经济合作区，推进跨境旅游合作区和防城边境经济合作区建设。加强与越南口岸通关合作，探索在东兴口岸实施“两国一检”监管新模式，建设“三个一”信息管理平台，完善国际贸易“单一窗口”。

　　四、加强港航交通设施建设，打造面向东盟区域性枢纽门户

　　强化港口龙头地位，加快构建综合交通体系，提升服务西南中南地区开放发展能力，凸显“一带一路”有机衔接门户作用。

　　抓好港口设施建设。按照专业化、现代化的要求，统筹大中小港口发展，加快渔万港区升级改造，推进企沙港区建设，打造北部湾国际航运中心。开工建设40万吨级码头及航道、企沙南防波堤，建成401号20万吨级大宗散货码头、501号化工码头和马鞍岭旅游码头等项目。到2020年，规划建设万吨级以上泊位43个，新建扩建航道79公里，港口货物吞吐能力达3.2亿吨，集装箱吞吐能力达300万标箱。

　　构建立体交通网络。加快防东铁路、防城港经上思至崇左铁路建设，推进南防线、云桂沿边铁路防城港段建设，建成疏港通道铁路支线和综合枢纽站场。推动钦防高速公路扩建，抓好东兴经上思至吴圩高速公路、东兴至峒中高等级公路规划建设，开工建设企茅一级公路，完善高速公路与城区路网连接布局，实现与周边城市1小时交通圈。加快民用航空建设，规划建设防城港支线机场，推进区县通用机场建设。

　　加快建成三大定位重要节点。按照中央赋予广西“国际通道、战略支点、重要门户”三大定位的要求，主动对接国家“一带一路”战略，完善以港口、铁路、公路、航空为主骨架的综合交通运输体系，构建面向东盟国际通道的重要枢纽；强化与西南、中南腹地的互动发展，推进产业合作，建成服务西南、中南地区开放发展的交通、旅游、金融、生态等支撑体系；更加突出我市在中国-中南半岛走廊的开放门户地位，建成中国-东盟产业合作先行基地和人文合作示范区。

　　五、着力提升城市功能，打造美丽宜居海湾城市

　　依托“三岛三湾”自然优势，优化城市空间布局，提升城市功能品位，建设靓丽海湾城市。

　　加快建设海湾新区。统筹中心区、防城江一江两岸、西湾新城规划建设，建成园博园，打造我市未来的政治文化中心、科技金融中心、现代服务业集聚中心和港航服务业配套中心。加快建设文昌新区、常山新区、江山大道以南等片区路网，建成伏波大道、江山大道西段等40多条道路，完善“五纵五横”城市交通网络。推进中心区供水厂、西湾污水处理厂、城市电力线路入地改造等工程。抓好海湾新区环海、环山景观亮化工程，建成环西湾40公里慢道，打造百里黄金海岸绿道。加快海绵城市建设，构建良好城市水生态体系，增强城市防洪防涝能力。完善公交、学校、医院、商务、市场、金融等配套，提升城市商圈，把海湾新区建设成为宜居宜业宜游宜学的现代化城区。

　　推进新型城镇化。加快县城和重点镇建设，培育沿海、沿边、沿江、沿线城镇发展轴。上思要围绕“山城氧都”的城市定位，加快南岸新区、江平新区开发建设，构建沿江建筑群和景观带。东兴要突出边关特色、民族风情、东盟元素，加快城市东扩北移，建设面向东盟的国门城市。推进企沙、江平、那良、在妙等示范镇建设，打造一批特色小城镇。推动产城深度融合，发展集生产、服务和消费于一体的产业新城。深化户籍制度改革，推进农业转移人口市民化，到2020年全市人口城镇化率达65%。

　　推进智慧城市建设。加快打造“数字防城港”，建成覆盖全市、统一集中的数据库及管理中心，推进城市管理信息化、数字化和智能化，提升城市管理效能。实施“宽带防城港”行动计划，推进智慧交通、智慧社区、智慧旅游等工程建设，实现主要公共场所免费WiFi全覆盖，农村宽带家庭普及率走在广西前列，让广大老百姓生活更方便、更舒适。

　　六、全力发展现代服务业，打造转型发展新优势

　　推动现代服务业提质增效，提高服务业竞争力，加快经济转型发展。

　　壮大现代物流业。建设现代物流平台，推动中外运东湾仓储物流基地、集装箱办理站、冷链物流等重点项目建设。抓好保税物流中心、东湾海公铁联运国家示范项目申建，创建国家现代物流创新发展试点城市。开通东北亚、东南亚集装箱直达航线，巩固防城港至昆明集装箱五定班列，增加防城港至北京、沈阳冷链专列密度，开通防城港至西安、成都等国内主要城市冷链专列，实现国际水果、海鲜、肉类海铁联运。

　　发展互联网经济。主动参与东盟信息港建设，发展大数据产业，建成北斗卫星一体化云计算、大数据应用中心。争取海底光缆、中越陆缆等项目落户防城港，建设辐射东盟的互联网服务平台。引进更多企业入驻“IT小镇”门户。创建跨境电商综合试验区，建好北投电商创业基地，推进防城港·阿里巴巴进口货源平台建设。抓好农村电商示范县（区）建设，建成一批“淘宝村”，打造全区电商创业示范基地。

　　培育金融服务业。发展新型金融业态，引进更多境内外金融机构入驻，构建多层次金融市场体系。推进人民币跨境业务创新，探索建立跨境人民币投融资中心、拆借中心，争取设立人民币投贷基金和外资股权私募基金，建立中国与东盟国家支付清算中心，开展离岸金融业务。

　　发展健康养老产业。加快建设功能完善、覆盖城乡的养老服务体系，建设一批滨海休闲、健康养老养生基地。探索养老服务产业社会化发展模式，鼓励民营资本建设医养结合养老机构，争创国家级医养结合试点城市。发展居家养老、社区照料和病患陪护等新型社区养老服务业态。发展健康养老地产，探索“养老养生+旅游休闲+房地产”新模式。

　　建设国际旅游目的地。加快完善江山半岛、京族三岛、十万大山、大东沙等景区景点设施，推进中越跨境旅游合作区和边海旅游带建设，创建一批4A级以上景区。建设国际邮轮母港、中越跨国旅游集散中心，抓好自行车专用道等一批旅游服务设施建设，开发滨海休闲度假、边境旅游、高山漂流、森林疗养、乡村旅游等特色旅游产品，建设一批特色旅游名村名镇。

　　七、推动城乡协调发展，打造全域防城港

　　实施全域统筹战略，增强农村经济发展活力，推进城乡基本公共服务均等化，缩小城乡差距。

　　统筹城乡协调发展。完善乡镇总体规划和土地利用规划，做好村（社区）建设规划，实现城乡规划“全域覆盖”。加快推进市域快速干道和村镇路网建设，推进村屯道路硬化，加快城乡公交一体化进程，构建城乡便捷交通体系，形成城乡交通“全域畅通”。建设农村社区一站式服务平台，加强农村公共文化体育服务，建设一批集中居住区文体广场，完善村镇医疗卫生服务，努力实现城乡基本公共服务“全域均衡”。

　　发展现代特色农业。实施现代特色农业产业“10+3”工程，推进现代特色农业示范区建设，建成自治区级示范区4个以上。推进双高糖料蔗基地建设，发展火龙果、澳洲坚果、金花茶等一批特色农产品。引进新品种新技术，发展水产健康养殖，实现渔业总产值200亿元。培育一批国家、自治区级农业产业化龙头企业。引导扶持一批农民合作社，发展家庭农场300个。加快农村产权制度改革，推动土地有序流转，鼓励发展股份合作。

　　推进“美丽家园”乡村建设。在巩固清洁乡村、生态乡村基础上，持续推进宜居乡村、幸福乡村建设。加快饮水安全、电网改造、通信网络、危房改造、排水排污等基础设施建设，健全农村基础设施运营管理长效机制。加强农村人居环境集中连片综合整治，创建绿色村屯、文明村镇，大幅提升农村人居环境质量。

　　八、实施创新驱动战略，打造创新型城市

　　主动适应新一轮产业变革挑战，改革公共政策供给方式，改造提升产业结构，构建多层次创新平台，培育发展新动力。

　　推进供给侧结构性改革。强化改革意识，坚持市场导向，加快行政管理、投融资、财税等领域改革，落实政府和社会资本合作项目以奖代补政策，建立PPP项目运作模式，落实增值税、消费税、资源税、环境保护和房地产税等改革措施，深化国资国企改革，帮助企业减少税费负担，降低成本。加快供销合作社综合改革。

　　推动创新产业集聚。实施知识产权战略行动计划和发明专利倍增计划，深化产学研合作，培育一批具有核心竞争力的高新技术骨干企业，打造创新型产业集群。抓好装备制造、生物医药、新能源、信息技术产业发展，培育一批超亿元新兴产业。创新人才引进机制，通过博士行和“白鹭英才计划”等柔性引才手段，吸引各类人才来防城港干事创业。

　　建设一批创新平台。加快高新技术开发区建设，促进科技资源和高新技术产业集聚，建成自治区级高新技术产业开发区。加强科技创新平台建设，引导支持企业自建或共建重点实验室、工程技术研究中心和企业技术中心等研发平台，建设各类研发平台30家以上。加快新产品、新技术的开发应用，建成科技企业孵化器、众创空间等创新创业服务平台10家以上。

　　九、推进共享发展，打造沿海最具幸福感城市

　　着力保障和改善民生，加快发展社会各项事业，不断增进民生福祉，建设和谐幸福防城港。

　　打赢脱贫教育卫生攻坚战。按照精准脱贫要求，统筹推进脱贫攻坚“十五大行动”，推进全市81个贫困村整村脱贫，在国家现行标准下，今年上思县要摘掉贫困帽子，2019年全市贫困人口实现脱贫。深化教育体制改革，抓好校长和教师队伍建设，促进基础教育、职业教育、高等教育全面发展。优化教育资源布局，补齐教育短板，抓好海湾新区等重点发展区域的教育基础建设，建成市北部湾高中，打造一所全国知名普通高中。提升广西财经学院防城港学院办学层次，争取把市理工学校建成面向东盟的国际职业技术学院。引进知名高校入驻，扩大我市高等教育办学规模。推进义务教育均衡发展，确保2020年前通过国家义务教育均衡发展验收。市第一人民医院建成三级甲等综合医院，完成市中医院、市妇幼保健院迁建，抓好中越友好医院和全科医生临床培养基地建设。完善社区卫生服务中心建设，推进城市公立医院综合改革，培养良好医德医风，建设一支素质过硬的医疗卫生队伍，齐心协力打造健康防城港。

　　完善社会保障体系。健全城乡居民养老和医疗保险制度，实现城乡养老、医疗保险全覆盖，完善城乡居民最低生活保障机制和社会救助制度，提高五保户、孤儿等弱势群体的供养水平，健全农村留守儿童和妇女、老人关爱服务体系。加强住房保障体系建设，抓好保障房建设和棚户区改造，到2020年农村危房要全部消除。促进就业创业，城镇登记失业率控制在4%以内。

　　建设边海文化名市。以公共文化服务标准化、均等化、社会化、数字化、制度化建设为基础，抓好市、县、乡、村四级公共文化设施网络建设。推进国学教育，开展全民读书活动，建设“书香港城”。加快创建国家公共文化服务体系示范区，建设边境民族地区公共文化服务体系，形成可推广、可复制的有效经验和做法，建成具有“五缘”特色的边海文化名市。开展全民健身活动，新建一批群众体育健身设施，抓好上思举重训练基地和东兴足球训练基地建设，推动各类体育场馆向公众开放。发展边山海特色文化，策划环海马拉松、自行车和汽车拉力赛等国际体育赛事，打造海上国际龙舟节、京族“哈节”等文化旅游节庆品牌。

　　加强生态保护。加强生态功能区和自然保护区管护，推进天然林保护、沿海防护林、生态公益林建设，创建国家森林城市、园林城市。加强城市环境保护，加大黑臭水体治理力度，推进城市污水垃圾处理项目建设，到2020年实现全市乡镇污水处理全覆盖，城市污水、垃圾无害化处理率达95%，主要江河流域水质达标率100%，城市空气质量优良率保持广西前列。实施“蓝色海湾”整治计划，保持良好海洋生态环境。

　　推进其他社会事业建设。创新社会治理，改进信访调解工作，完善群众诉求表达机制，依法调处化解各种矛盾纠纷。健全重大决策社会稳定风险评估制度。实施“三网四联五达标”工程，建设幸福社区。抓好人口计生工作，积极发展妇女儿童、残疾人事业。支持非公有制经济发展，激发民营经济活力。加强食品药品安全监管，建成市食品药品检验检测中心，完善生产安全和社会治安防控体系，健全防灾减灾救灾体制，筑牢港城安全网。

　　各位代表！边疆的稳固安定关系全市发展大局，必须要深入实施爱边富民战略，加强边海防、社会治安防控，加强全民国防教育和国防动员建设，推动军民融合深度发展，保持打击走私高压态势，深化公安改革，加强反恐、禁毒等国际警务协作，提高应急救援能力，打造法治防城港、平安防城港，维护边疆安宁。

　　十、加强政府自身建设，打造良好营商环境

　　建设法治政府。着力提升依法行政水平，把政府工作全面纳入法治轨道。完善行政决策程序，重大行政决策充分听取群众意见，推进政府依法科学决策。自觉接受人大的法律监督、工作监督，把政治协商、民主监督纳入决策程序。建立政府法律顾问制度，健全公共法律服务体系。强化行政执法监督，重视行政监察、审计监督和社会舆论监督。

　　建设效能政府。强化政府效能建设，加大简政放权、放管结合、职能转变力度，优化行政审批流程，减少审批事项。完善政府权力清单、责任清单和负面清单，强化事中事后监管，抓好“两随机一公开”工作。创新政府服务管理，推进网上审批，提升运行效率；整合乡镇“七站八所”政务服务资源，实现政务服务便捷化，打通群众办事“最后一公里”。整治慵懒散等不良风气，克服形式主义、官僚主义，不断提高政府执行力。

　　建设廉洁政府。坚持勤俭办事，事事精打细算，处处严格把关。加强国有资产监管，构建科学有效的监管机制。全面推进预决算公开，让公共资金在阳光下运行。强化重点领域和关键岗位廉政风险防控，严格规范权力行使，加强经济责任审计，从严治政，克己奉公，树立政府清正廉洁的良好形象。

　　各位代表，时代赋予重托，奋斗铸就辉煌！未来五年，防城港市发展前景更加美好，改革发展任务更加繁重。让我们紧密团结在以习近平同志为总书记的党中央周围，在市委的坚强领导下，齐心协力，求真务实，开拓进取，为加快实现广西“两个建成”先行区目标而努力奋斗！
